# Ultrastructure and 3D reconstruction of a diplonemid protist (Diplonemea) and its novel membranous organelle

**DOI:** 10.1128/mbio.01921-23

**Published:** 2023-09-22

**Authors:** Daria Tashyreva, Jiří Týč, Aleš Horák, Julius Lukeš

**Affiliations:** 1 Institute of Parasitology, Biology Centre, Czech Academy of Sciences, České Budějovice, Czech Republic; 2 Faculty of Sciences, University of South Bohemia, České Budějovice, Czech Republic; Duke University, Durham, North Carolina, USA

**Keywords:** Euglenozoa, cell division, ultrastructure, 3-dimensional reconstruction, diplonemid, SBF-SEM

## Abstract

Although diplonemid protists (Diplonemea, Euglenozoa) are among the most species-rich microeukaryotes in the ocean, many ultrastructural features of this lineage remain to be clarified. Using serial block-face scanning and transmission electron microscopy, we provide the whole-cell three-dimensional model of a previously undescribed diplonemid *Lacrimia vacuolata* sp. nov. For the first time, we were able to reconstruct a diplonemid cell in detail, including all its organelles and the flagellar and feeding apparatuses. By analyzing various stages of the cell cycle, we provide the first description of a diplonemid cell division, which is characterized by the disassembly of flagellar axonemes and the feeding apparatus and their further construction in the daughter cells. Moreover, we have identified a novel, ultrastructurally complex organelle, herein named the colv (Center for Organization of Layered Vesicles). We suggest that the colv is involved in food processing and membrane trafficking, and describe its close association with other components of the cellular digestive system.

**IMPORTANCE:**

The knowledge of cell biology of a eukaryotic group is essential for correct interpretation of ecological and molecular data. Although diplonemid protists are one of the most species-rich lineages of marine eukaryotes, only very fragmentary information is available about the cellular architecture of this taxonomically diverse group. Here, a large serial block-face scanning electron microscopy data set complemented with light and fluorescence microscopy allowed the first detailed three-dimensional reconstruction of a diplonemid species. We describe numerous previously unknown peculiarities of the cellular architecture and cell division characteristic for diplonemid flagellates, and illustrate the obtained results with multiple three-dimensional models, comprehensible for non-specialists in protist ultrastructure.

## INTRODUCTION

Diplonemea, or diplonemids, is a lineage of heterotrophic flagellates belonging to the phylum Euglenozoa. Because of their inconspicuous morphology, diplonemids have long remained overlooked. Moreover, due to the unusually long V4 region of their 18S rRNA, their diversity has been significantly underestimated in metabarcoding studies (1). However, recent evidence of extreme molecular species diversity and abundance of diplonemids in the ocean (1 – 6) has attracted increased attention to this understudied group of protists. Additionally, the improvement of isolation and cultivation techniques (7, 8) has allowed for the descriptions of new species and genera (9).

In the past decade, diplonemids have become known for their unusual cell and molecular biology, such as large nuclear genomes rich in genes as well as repetitive sequences (3, 10, 11), extraordinary complexity of mitochondrial genomes and transcriptomes (12, 13), endosymbiotic associations with a range of bacteria (14, 15), complex life cycles (9), a sophisticated endomembranous system (16), and the ability to accumulate large quantities of inorganic crystals (17). Moreover, one species, *Paradiplonema papillatum*, became a genetically tractable model allowing for detailed dissection of diplonemid molecular and cell biology (18). At the same time, many ultrastructural features of diplonemids remain to be clarified. So far, detailed studies have focused mostly on the ultrastructure of the flagellar and feeding apparatuses (19 – 22). However, a targeted detailed ultrastructural characterization of an entire diplonemid cell and peculiarities of its division are still missing.

In this work, we describe a new diplonemid, *Lacrimia vacuolata* sp. nov., only a second named member of a relatively large clade so far comprised of environmental sequences (8). Moreover, we provide the first three-dimensional (3D) reconstruction of a diplonemid cell. This protist has been selected for 3D modeling due to the presence of a novel organelle and an exceptionally rich endomembranous system, which includes vesicles and vacuoles of various size and content, in addition to conventional membrane-trafficking structures, such as the endoplasmic reticulum (ER) and Golgi apparatus (GA). Here, we provide a detailed morphological characterization of this novel organelle and discuss its possible functions.

## RESULTS

### Isolation and molecular phylogeny

**Fig 1:**
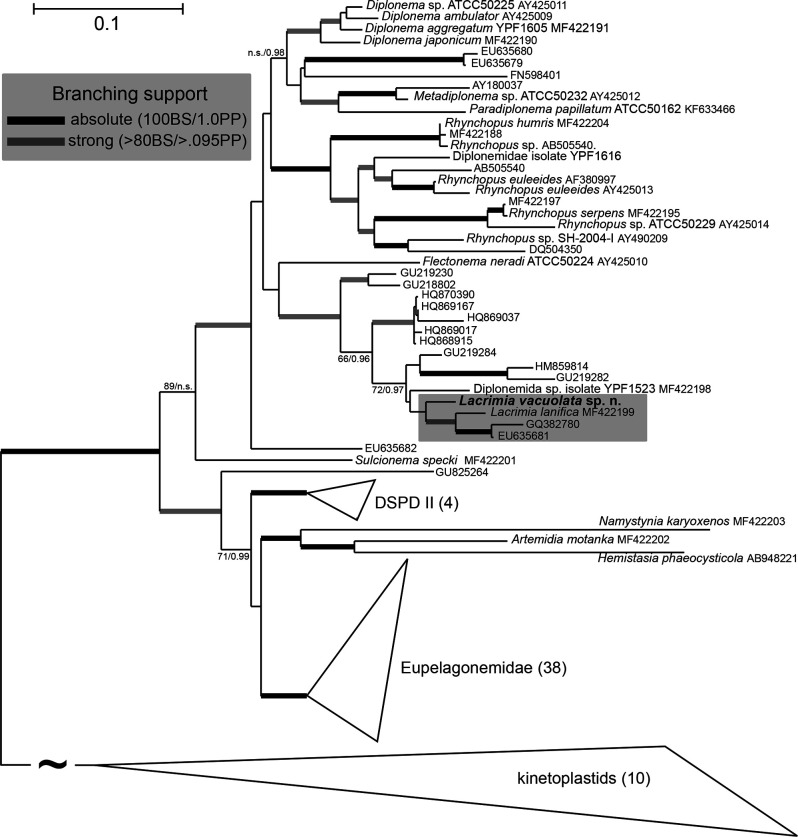
Maximum-likelihood 18S rDNA phylogeny of *Lacrimia vacuolata* sp. nov. in the context of known diplonemid diversity. The tree was reconstructed using IQTree 2 under the general time-reversible evolution model with empirical nucleotide frequencies estimated from the data set and four relaxed rate categories (GTR + F + R4). The topology is based on the analysis of 1,925 unambiguously aligned sites of 86 diplonemids and 10 kinetoplastids used as an outgroup. Branching support (categorized either by thickened branches or shown by numbers) is represented by non-parametric bootstraps (BS) estimated from 500 replicates in IQTree 2 under the conditions described above and by Bayesian posterior probabilities (PP). The latter were estimated in Phylobayes 4.1 from four MCMC chains run under the GTR model in combination with 20 empirical mixture profiles (GTR + C20) until they converged. Only branching support with either BS >80 or PP >0.95 are shown; n.s. = unsupported. To improve the readability of the ingroup topology, the length of the branch leading to the outgroup clade was shortened by ~75%.

The strain YPF1808 was isolated into a stable axenic culture from a planktonic sample originating from the Sea of Japan. Based on the divergence of its 18S rRNA from previously sequenced diplonemids, the YPF1808 isolate is designated here as a new species, named *Lacrimia vacuolata* sp. nov. Maximum likelihood phylogeny of the 18S rRNA alignment containing 96 taxa and 1,925 sites yielded a topology congruent with previous studies (Fig. 1) (14, 23). Rooted with kinetoplastids, hemistasiids, and eupelagonemids, Diplonemidae constitute several divergent basal-branching sequences, represented by the genera *Sulcionema* and *Flectonema*, as well as three well-defined clades at the crown of the tree. These are recognized as (i) *Diplonema* s.l., recently split into the genera *Diplonema*, *Metadiplonema,* and *Paradiplonema;* (9) (ii) the genus *Rhynchopus*; and (iii) an operational taxonomic unit (OTU)-rich clade containing 12 environmental sequences, an undescribed isolate YPF1523 and two *Lacrimia* species, one of which is *L. vacuolata* sp. nov. described here (Fig. 1).

Within this third clade, the two *Lacrimia* species grouped together with mesopelagic environmental OTUs; however, the branching order within this subclade lacked support. Therefore, we have created an alternative topology with forced polyphyly of *L. vacuolata* and remaining representatives of the *Lacrimia* clade (*L. lanifica* and two “environmental” sequences) and compared its log-likelihood scores with the original topology using the approximately unbiased test as implemented in Consel. Based on these results, we were able to reject this alternative topology (*P* < 0.05). Moreover, the inclusion of herein described diplonemid into the genus *Lacrimia* is also supported by basic DNA statistics. While the average 18S rRNA-based similarity of *L. vacuolata* sp. nov. to species of the *Diplonema* s.l. clade and *Rhynchopus* spp. is 85.9% and 83.5%, respectively, the similarity of *Lacrimia lanifica* reaches 92.9% and for the unnamed diplonemid YPF1523 92.1%. A more detailed overview of 18S rRNA similarities within Diplonemidae (and Hemistasidae) is shown in Table S1, with a taxonomic summary of *L. vacuolata* available as Text S1.

### Light and fluorescence microscopy

**Fig 2:**
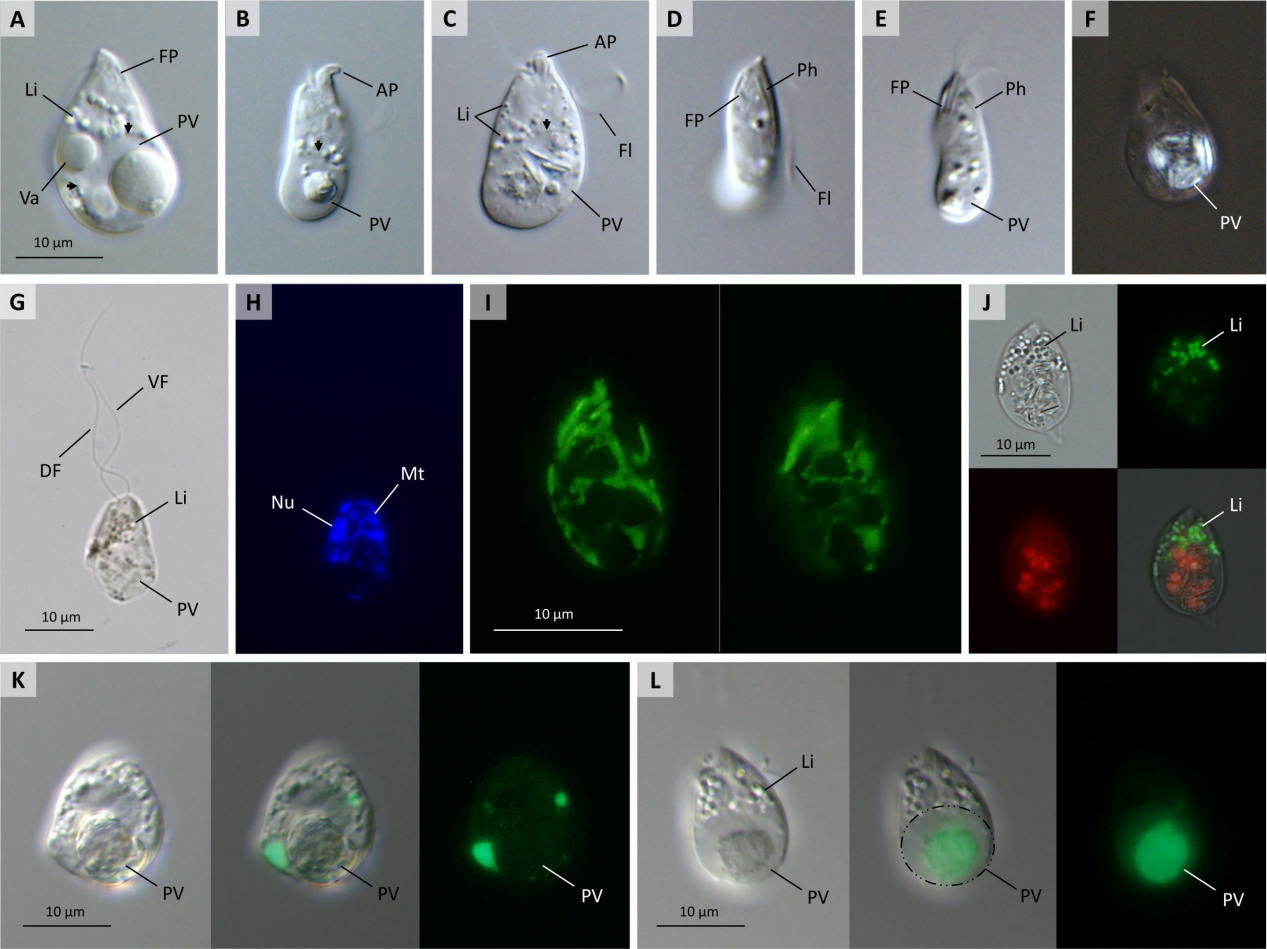
Differential interference contrast micrographs of *Lacrimia vacuolata* sp. nov. (**A–E**). Live cells with teardrop morphology (**A and C**) and cylindrical shape (**B and D**) showing variable-sized PV delimited in (**A–C) **with arrows, AP, smaller vacuoles (Va), deep FP, and cytopharynx (Ph), flagella (Fl), and lipid-like cytoplasmic inclusions (Li). Live cell viewed under polarized light (**F**) showing needle-like crystals. OsO_4_-fixed cell (**G and H**) with subequal dorsal (DF) and ventral flagella (VF) and showing 4,6-diamidino-2-phenylindole-stained nucleus (Nu) and mitochondrial DNA (Mt). Images of live cell at different focal planes, stained with SYTO24 (**I**), show reticulate distribution of mitochondrial DNA. Lipid droplets stained with BODIPY in live cell (**J**). Live cell with LysoTracker-stained smaller acidic compartments (**K**) and larger pellet inside posterior vacuole (**L**) delimited by circle.

Light microscopic examination of freshly inoculated cultures revealed cells that are variable in size, measuring from 10.1 µm to 23.4 µm in length (18.1 ± 3.1 µm; *N* = 50) and 5.9 µm to 12.9 µm in width (9.5 ± 2 µm; *N* = 50). Bigger cells are typically teardrop- or pear-shaped due to the narrowed anterior and the presence of a large posterior vacuole (PV), which is often accompanied by an additional one to three smaller vacuoles that collectively occupy up to two-thirds of the cell volume (Fig. 2A and C). The smallest cells have rather cylindrical bodies, constricted anteriorly, and always contain a smaller posterior vacuole (Fig. 2B and E). These vacuoles are variable in opacity and texture (Fig. 2A, C, K, and L), and often carry large brownish or colorless granules and long needle-like crystals that prominently polarize light (Fig. 2F). The anterior half usually contains numerous small lipid droplets (Fig. 2A, L, and J). A characteristic J-shaped cytopharynx, apical papilla (AP), and deep flagellar pocket (FP) can be readily seen at the light microscopy level (Fig. 2B through E). In older batch cultures, cells become generally smaller and often immobile, but do not differentiate into other life stages, such as fast-swimming cells, cysts, or sessile cells.

The flagella are parallel to each other, arise subapically from a deep FP (Fig. 2E), and measure one to two times the length of the body. They are similar in width but subequal in length—the anterior or dorsal flagellum (DF) is usually one-third longer than the posterior or ventral flagellum (VF) [Fig. 2G; serial block-face scanning electron microscopy (SBF-SEM) data]. Relatively slow forward swimming, during which the cell continuously oscillates around its longitudinal axis, is mediated by the DF that forms a large loop around the anterior half and the VF that is loosely wrapped along cell body (Movie S1 ). Forward swimming is often interrupted by rotational movements. During occasional backward swimming, both flagella are straight and parallel to each other and directed anteriorly. Movements, similar to metaboly in euglenids, are manifested as twisting and contraction and expansion of the entire body (Movie S1). Cells are also capable of gliding along surfaces by touching them with the anterior tip and aiding the propulsion with high-frequency but short-amplitude waving of both flagella (Movie S1).

The nucleus is subspherical and localized peripherally in the anterior half of the cell (Fig. 2H). Vital staining of mitochondrial DNA with nucleic acid stain SYTO24 revealed peripheral localization and reticulated structure of the mitochondrion (Fig. 2I). To shed light on the nature of the posterior vacuoles, live cells were incubated with LysoTracker, a pH-sensitive dye staining acidic cellular compartments such as autophagosomes, lysosomes, and digestive vacuoles (24). A few brightly stained medium-sized vacuoles were localized in the anterior half of the cell or around the large posterior vacuole (Fig. 2K), which itself did not show uniform staining. While the whole volume of the posterior vacuole was not acidified, it contained one or several smaller acidic pellets (Fig. 2L), which likely appeared as a result of its fusion with smaller digestive vacuoles. Additionally, several small dots could be often seen in the cell anterior (data not shown).

### Ultrastructural characterization

#### Cytoskeleton, feeding, and flagellar apparatuses

**Fig 3 F3:**
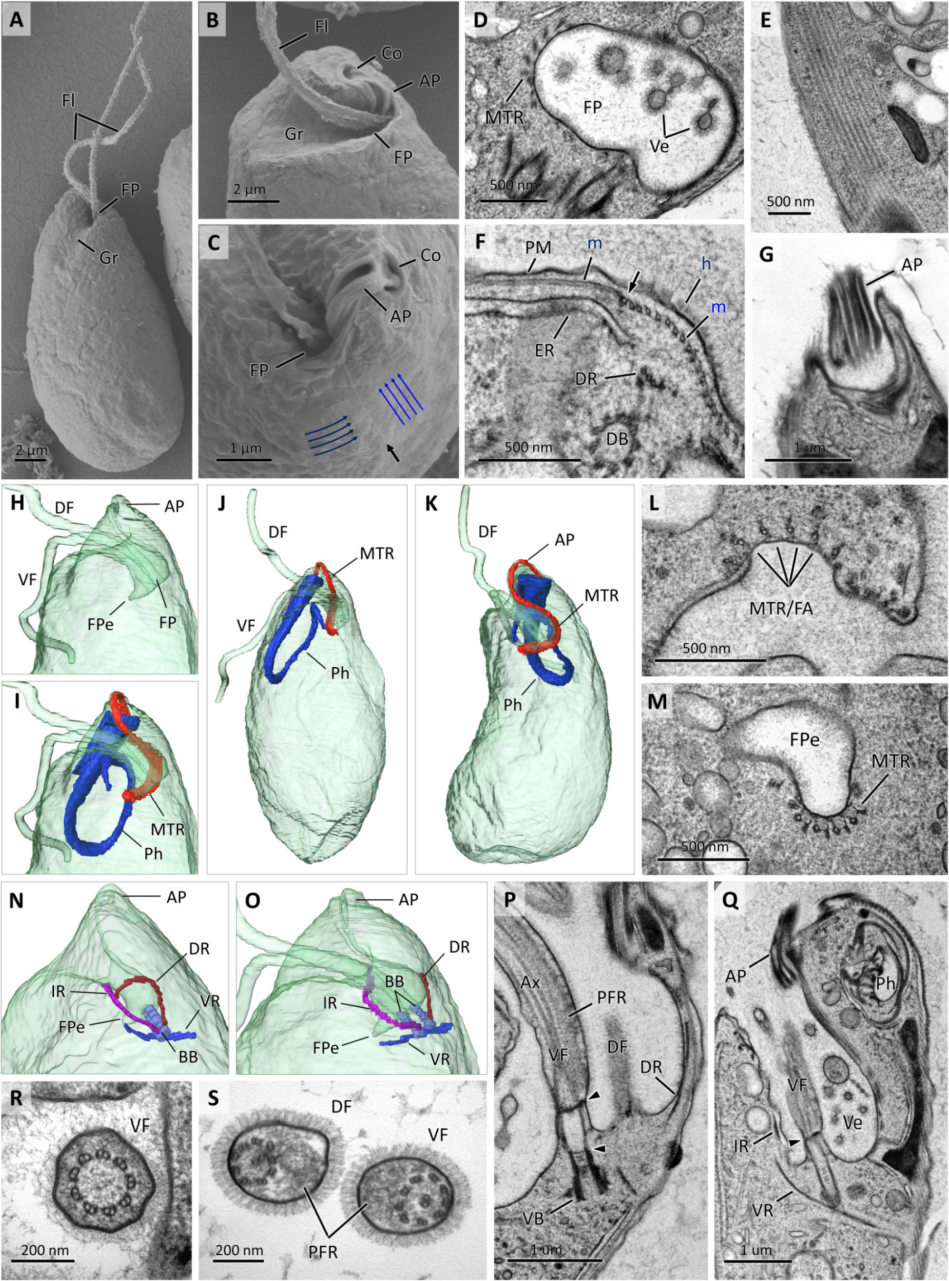
Scanning electron micrographs (**A–C**) of *Lacrimia vacuolata* sp. nov. General appearance of cell (**A**) showing flagella (Fl), anterior groove (Gr) and entrance to FP. Apical part of cells (**B and C**) additionally displays rigid collar (Co) encircling cytostome and AP. Note orientation of corset microtubules marked by blue arrows and junction seam labeled with black arrow. Transmission electron micrographs (TEMs) of cell apex and cytoskeleton (**D–G**). Oblique section through FP (**D**) with dense hair-coated lining and hair-coated vesicles (Ve) budding off FP membrane. Microtubular corset (**E**). Transverse section through base of FP (**F**) showing junction point (arrow) of perpendicularly oriented microtubules (M), PM covered in hair (H), and ER underlying microtubular corset. Section through microtubule-reinforced AP (**G**). Surface rendering (SR) view of cell reconstructed from SBF-SEM data set (**H–K**) showing relative position of FP and FP extension (FPe), DF and VF, reinforcing microtubules (MTR) supporting FP and AP, and cytopharynx (Ph). TEM of cross-sectioned FP (**L**) and FPe (**M**); note MTR attached to FP membrane and four microtubules continuous with feeding apparatus (MTR/FA). SR view of two cells (**N and O**) illustrating position of basal bodies (BB), dorsal (DR), ventral (VR), and intermediate roots (IR). Longitudinal TEM sections through FP (**P and Q**); note transitional zone of flagellum between transitional plates (arrowheads), axoneme (Ax), paraflagellar rod (PFR), and hair-coated Ve. Transverse TEM sections (**R and S**) through transitional zone of flagellum (**R**) and axoneme (**S**) supported with PFR with tubular lattice in DF and parallel lattice in VF.

**Fig 4 F4:**
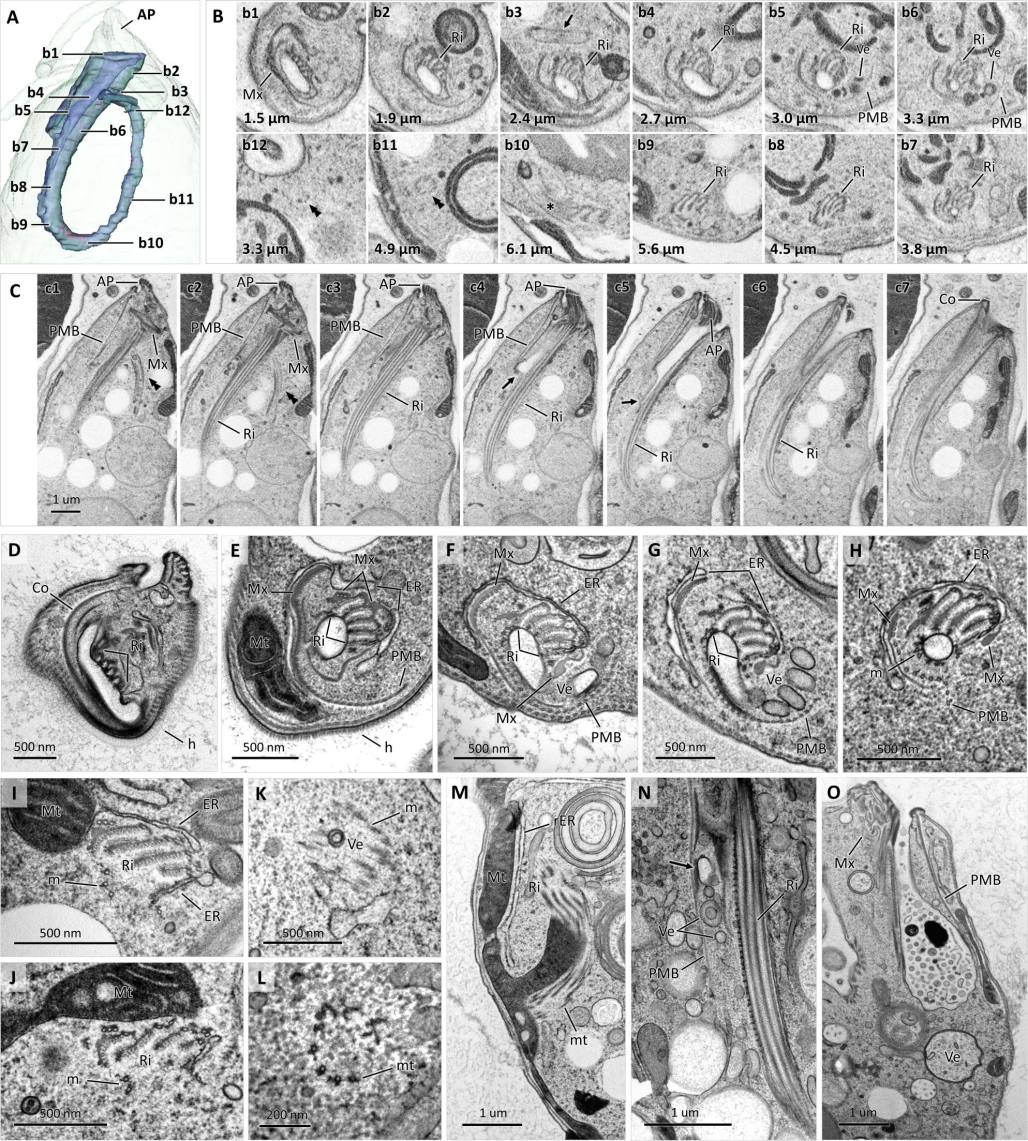
Ultrastructure of feeding apparatus. Semi-transparent SR view of cell apex reconstructed from SBF-SEM data set (**A**) illustrating cell surface including AP and surface and lumen of cytopharynx. Cross-sections of cytopharynx in non-sequential SBF-SEM slices (**B**); images **B1–B12** correspond labeling in **A**; length in micrometers indicates depth into cell from tip of AP; note presence of supporting ribs (Ri) in **B2–B9** and their disappearance in **B10** at upward turn (asterisk), peripheral microtubular band (PMB) directing exit of Ve in **B5 and B6**, and microtubular circle in ascending part of cytopharynx (**B11**) and its descending part after second turn (**B12**), both labeled with double arrowheads. Sequential SBF-SEM images (**C**) taken at 100 nm steps showing longitudinally sectioned feeding apparatus; note collar (Co) at distal end of cytopharynx. Cross TEM sections of cytopharynx (**D–L**) through Co (**D**) and parts approximately corresponding to **B2** (**E**), **B4** (**F and G**), **B7** (**H**), **B9** (**I**), **B10** (**J**), **B11** (**K**), and **B12** (**L**); entire descending part (**D–J**) is half-encircled with smooth ER; m points at microtubules; Mx points at matrix; Mt labels mitochondria. Mitochondrion-cytopharynx junction at its upward turn (**M**); rough ER (rER) underlies mitochondrion branch. Longitudinal section through cytopharynx showing exiting round- and ring-shaped small Ve (**N**) and formation of large Ve budding of dilated cytopharyngeal lumen (**O**).

The cell apex is asymmetrical, due to the subapical position of the FP, which is located in a pronounced depression that merges into a spiral groove (Fig. 3A and B). The cell surface is smooth (Fig. 3A through C) and surrounded by a naked plasma membrane (PM), beneath which a peripheral corset of densely packed, interconnected, parallel microtubules can be seen (Fig. 3E and F). The microtubules helically wrap around the entire cell, some of which terminate at the level of FP, on the side opposite the spiral groove by making a perpendicular junction with vertically oriented microtubules (Fig. 3C and F). The entire cell apex, including the FP lining and both flagella, is densely covered with fine, non-tubular hairs (Fig. 3D, F, S and 4D, E). Numerous small vesicles branching from the FP membrane into the extracellular space are also coated with these hairs (Fig. 3D and Q).

The cytostome, which opens apically, is associated with a narrow lip-like protrusion of cytoplasm called papilla and encircled with an elongated rigid collar (Fig. 3B, C, and G). The cytostome descends as a tubular cytopharynx at an angle of 30° relative to the longitudinal axis of the body, more or less parallel to the cell surface (Fig. 3I and J). It makes a U-turn approximately at one-third the distance into the cell (~6–7 µm), ascends until it measures 3/4 of the length of the descending part, and then makes an additional turn downward (Fig. 3I through K and 4A [Fig F3 F4] [Fig F3 F4]; Movie S3). The FP opens ~1.5 µm–2 µm below the cytostome, is approximately 2.5 µm deep, descends at an angle of 45° relative to the cell’s longitudinal axis, and positioned in the opposite orientation to the cytopharynx (Fig. 3A, H, and N). A funnel-like, lateral extension of the FP is directed to the cell center (Fig. 3H, N and O).

The general architecture and complexity of the feeding and flagellar apparatuses is similar to that of freshwater *Diplonema* (20, 21). The cytopharynx and FP are connected by a distinct band of parallel microtubules. It originates from the terminus of the FP extension (Fig. 3I through K and M), ascends along the proximal part of the FP wall, loops around the apical papilla forming its ridges (Fig. 3G) and finally descends along the pharyngeal lumen (Fig. 3I through K) to become a part of the cytopharynx-reinforcing ribs (see below). Within the flagellar pocket, this supporting microtubular band has been termed “reinforcing microtubules” (MTR) (25). It is composed of several microtubules, only four of which are continuous with the cytopharynx. The MTR microtubules are embedded into electron-dense matrix that connects them to each other and to the proximal side of the FP wall (Fig. 3L and M) as well as to the plasma membrane covering the papilla. In addition, many solitary microtubules, projecting in different directions, can be seen at the bottom of the FP and around its extension (Fig. 3Q).

Flagella arise from basal bodies oriented parallel to each other (Fig. 3F and O). The dorsal basal body is connected to a single microtubular dorsal root, which originates on the outer side of the dorsal basal body and ascends along the FP wall (Fig. 3N, O and P). The ventral basal body is distinguished by asymmetrically arranged ventral and intermediate roots. The latter originates on the interior side of the ventral basal body and, like the dorsal root, ascends along the FP wall (Fig. 3N and O; Movie S2). Although both the dorsal and intermediate roots spiral around the FP, the exact point of their termination is difficult to determine since they eventually become indistinguishable from microtubules of the subpellicular corset (Movie S2). The ventral root is laterally anchored to the outer side of the ventral basal body and oriented perpendicularly to the cell’s longitudinal axis (Fig. 3N, O and Q; Movie S2). The ventral root originates near the microtubular corset and either terminates at the terminus of the FP extension or is continuous with the MTR (Fig. 3N).

Between the axonemes and basal bodies lies a transitional zone of the flagellum, which is delimited by thin proximal and thick distal cross-plates (Fig. 3P and Q), and has a configuration of nine outer doublets (Fig. 3R). The axonemes of both flagella invariably consist of two central microtubules surrounded by nine outer doublets, a typical arrangement for a eukaryotic flagellum (Fig. 3S). Although the flagella are morphologically indistinguishable at the light microscopy level, transmission electron micrograph (TEM) showed that the dorsal flagellum bears a smaller paraflagellar rod (PFR) with a tubular lattice, whereas the ventral flagellum is accompanied by a thicker PFR with a parallel lattice (Fig. 3S). In both flagella, the PFR is attached to the distal transitional plate, which is located slightly above the insertion point of the flagellum (Fig. 3P).

The cytopharynx is formed by an array of supporting elements, which includes ribs, several bands and clusters of microtubules, and a matrix associated with them. The composition of these elements varies throughout its length. In its most distal part, the cytopharynx is surrounded by a semi-circle of microtubules called the peripheral microtubular band (PMB) embedded in a dense matrix continuous with the rigid collar around the cytostome (Fig. 4C through E). As the cytopharynx descends, the matrix becomes thinner and separates into several elements forming flat vertical rods associated with additional bundles of microtubules (Fig. 4E and F). Simultaneously, an ensemble of four ribs assembled into a partial rosette begins to emerge (Fig. 4B; Movie S3). These are formed by fivefolds anchored with microtubules that are continuous with the MTR (Fig. 4E through H). The ribs further elongate, and most of the matrix gradually disappears, except for small portions associated with the PMB and the ribs (Fig. 4F and G). Three to five microtubules, embedded into electron-dense material resembling the MTR, attach to the pharyngeal lumen membrane (Fig. 4F through H). Approximately 3 µm down the cell, numerous round, elongated, and ring-shaped vesicles start to pinch off the pharyngeal lumen, which are directed away by the PMB (Fig. 4B, F, G and N; Movies S2 and S3). The pharyngeal lumen, which had nearly the same diameter for its entire length, now tapers sharply and then terminates completely (Fig. 4A through C). In addition, the cytopharynx appears to have the ability to stretch to engulf larger particles (Fig. 4O). In the tapering region, the PMB gradually disappears, but ribs and small clusters of microtubules continue to descend (Fig. 4B, I and J). Unlike in *Diplonema* spp., no additional bundles of quadratically packed microtubules appear at the proximal part of the cytopharynx (9). For most of its downward length, the descending part of the cytopharynx is half-encircled with smooth ER (Fig. 4E through I). The ribs become highly reduced and eventually disappear at the proximal end, where the cytopharynx forms a U-turn and makes contact with a mitochondrial branch. (Fig. 4B, J and M). The cytopharynx continues to expand in the ascending part as a circle of microtubules (Fig. 4A, B and K) and reduces to a semi-circle of five microtubules after the downward turn (Fig. 4A, B and L). The area, delimited by these microtubules, is filled with ribosomes, although numerous small vesicles (50 nm–100 nm in diameter) are often seen in the ascending part (Fig. 4L). The cytopharynx terminates as the remaining microtubules disband and radiate into the cytoplasm in different directions (Movie S2). Tubular extrusomes, typical for many diplonemids and other euglenozoans, were absent in *L. vacuolata*.

#### Cell division

**Fig 5:**
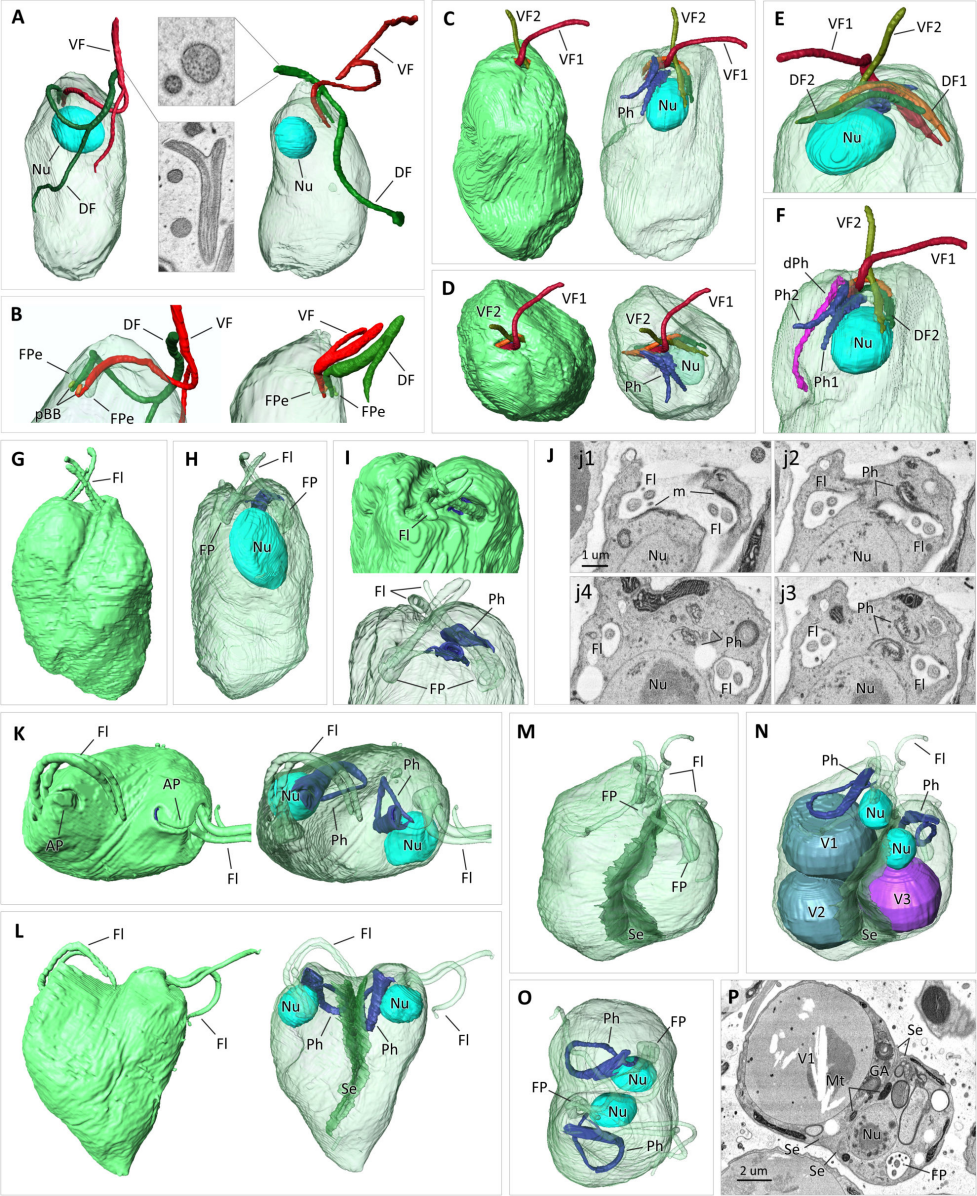
Cell division of *L. vacuolata* sp. nov. SR view of cells reconstructed from SBF-SEM data set (**A–I and K–O**). Side view of two pre-division cells with kink in both flagella (**A**) and side view of cell apexes with flagella severed at one (left) or multiple (right) sites (**B**); note the presence of two FPe and pro-basal bodies (pBB). Side (**C**, **E**, and **F**) and top views of cell (**D**) at earlier stage of division displaying elongated nucleus (Nu) closely associated with flagellar-cytopharyngeal complex; note absence of apical papilla (**C and D**), two nascent cytopharynxes (Ph1 and Ph2) in **C**, **D,** and **F** and degrading maternal cytopharynx (dPh) illustrated in **F**; both dorsal flagella (DF1 and DF2) are concealed inside extended flagellar pocket, while both ventral flagella (VF1 and VF2) protrude beyond flagellar pocket. Side (**G and H**) and top (**I**) views of cell with both pairs of flagella (Fl) beyond nearly segregated FP without apical papillae and with elongated pre-division Nu. Non-sequential SBF-SEM sections (**J**) through FP-Ph complex of cell depicted in **G–I**; note dark microtubular band stretched along FP in **H1** that forms two new Ph in **J2 and J3** and vesiculated appearance of Ph at their proximal part (**J4**). Top (**K**) and side (**L**) view of cell that underwent nuclear (Nu) division, with two fully formed Ph and AP. Cell at late stage of division (**M–P**) with septum (Se) separating nascent daughter cells of subequal size with different number of vacuoles (**V1, V2, V3**); note Nu (**N and O**) and other organelles (**N**), including GA aligned along V-shaped Se.

The earliest sign of the onset of cell division is a notable enlargement of the nucleus and nucleolus followed shortly by the duplication of basal bodies (Fig. 5A and B). Most of these cells also exhibit either one or both flagella with a kink formed by severing of the axoneme under the flagellar membrane (Fig. 5A and B). The axonemes were often fragmented into several segments that seemingly moved under the flagellar membrane, as two to four such segments were seen in the cross-sectioned flagella. As the division progresses, the basal bodies, all now bearing flagella, usually start to migrate to opposite sides of the cell. It appears that the daughter cells do not inherit a fully grown flagellum from the parental cell. Instead, both receive a pair of short flagella (Fig. 5C, G, and L), indicating prior disassembly or shedding of axonemes and their further construction at later stages of cell division (Fig. 5M). Initially, both new dorsal flagella are fully concealed inside the FP, whereas ventral flagella protrude beyond the FP (Fig. 5C through F). At this point, the ventral roots cannot be seen. The normally subspherical nucleus grows bigger and attains an ellipsoid shape (Fig. 5E and H), and the nucleolus further enlarges. Through the entire cell division, the nuclei stay in close contact with the FP-pharyngeal complex (Fig. 5E, H, K, and O) and the nuclear envelops remain intact. The cytopharynx of the maternal cell degenerates, and the papilla is no longer visible (Fig. 5C and D). The microtubules that supported it are now stretched along the apical part of the FP (Fig. 5J). While the ribs from the degenerated maternal cytopharynx are still present within a dividing cell (Fig. 5F), two new cytopharynxes form as invaginations in twofold rotational symmetry of this expanded FP (Fig. 5J).

As the division progresses, both cytopharynxes move apart and elongate, and their growing proximal regions acquire a vesicular appearance (Fig. 5J4). All four flagella elongate and emerge from the FP (Fig. 5G through I); however, the ventral roots are not yet present. Subsequently, the newly formed nuclei either migrate to the opposite sides of a dividing cell following their respective FP-pharyngeal complexes (Fig. 5K and L) or remain adjacent to the septum along with them (Fig. 5N and P). After the nuclei and FP-pharyngeal complexes segregate into nascent daughter cells, a septum begins to form at the anterior end of the cell, separating them from each other (Fig. 5L).

Before cytokinesis is complete, cytopharynxes, papillae (Fig. 5M and O), and all flagellar roots are fully formed. The septum separating the daughter cells is formed along the entire length of the dividing cell before the cleavage furrow becomes apparent (Fig. 5M). This septum is formed by invaginations of the microtubular corset, each outlining nascent daughter cells, between which on each side lies a cytoplasmic V-shaped bridge (Fig. 5M and P). At this stage, all organelles are segregated into the daughter cells, and the mitochondria are aligned along the septum consistent with their typical peripheral position in a diplonemid cell (Fig. 5P). The binary fission of *L. vacuolata* may result in subequal daughter cells that considerably vary in size and receive different numbers of large vacuoles (Fig. 5M and N).

#### Nucleus, mitochondrion, ER, and Golgi apparatus

**Fig 6 F6:**
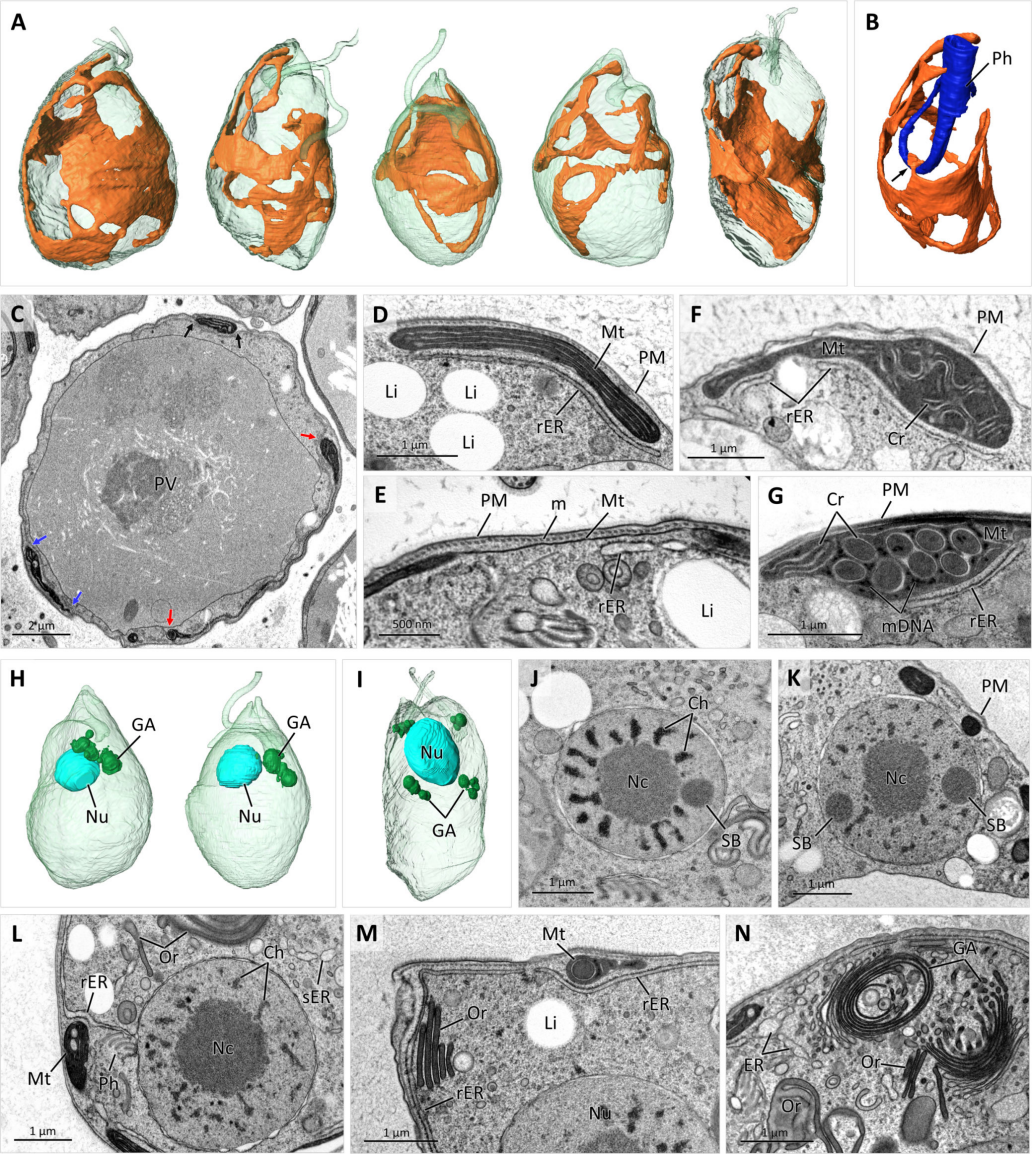
Morphology and ultrastructure of mitochondrion and nucleus. Surface rendering view of five cells (**A**) reconstructed from SBF-SEM data set, each displaying single reticulated mitochondrion and illustration (**B**) of mitochondrion to cytopharynx (Ph) junction point marked with arrow. SBF-SEM image (**C**) of cell cross-sectioned through PV; spaces between arrows indicate mitochondrial branches. TEM images (**D–G**) of peripheral mitochondria (Mt) localized under PM and microtubular corset (**M**) and showing long parallel stacks of lamellar cristae (**D**), curved short cristae (Cr) in **F**, circular profiles of unusual tubular Cr (**G**) with DNA aggregates (mDNA) between them; thicker branches connected with thin bridges (**E**) devoid of cristae; sacs of rER are localized below Mt; lipid inclusion indicated as Li. Three cells reconstructed from SBF-SEM data set (**H and I**) with subspherical anterior nuclei (Nu) and perinuclear Golgi bodies (GA); dividing cells (**I**) contain multiple small GA. TEM images of Nu (**J-L**) sectioned through large central nucleolus (Nc) and peripheral condensed chromosomes (Ch) and spherical bodies (SB); peripheral rER is continuous with outer nuclear membrane and cytopharynx-associated (Ph) ER (**L**); smooth ER (sER) is localized in central cytoplasm (**L**). TEM images (**M and N**) illustrating extensive sacs of rER under PM and over Mt branches (**M**) and GA composed of multiple stacked sacs and vesicles (**N**).

**Fig 7:**
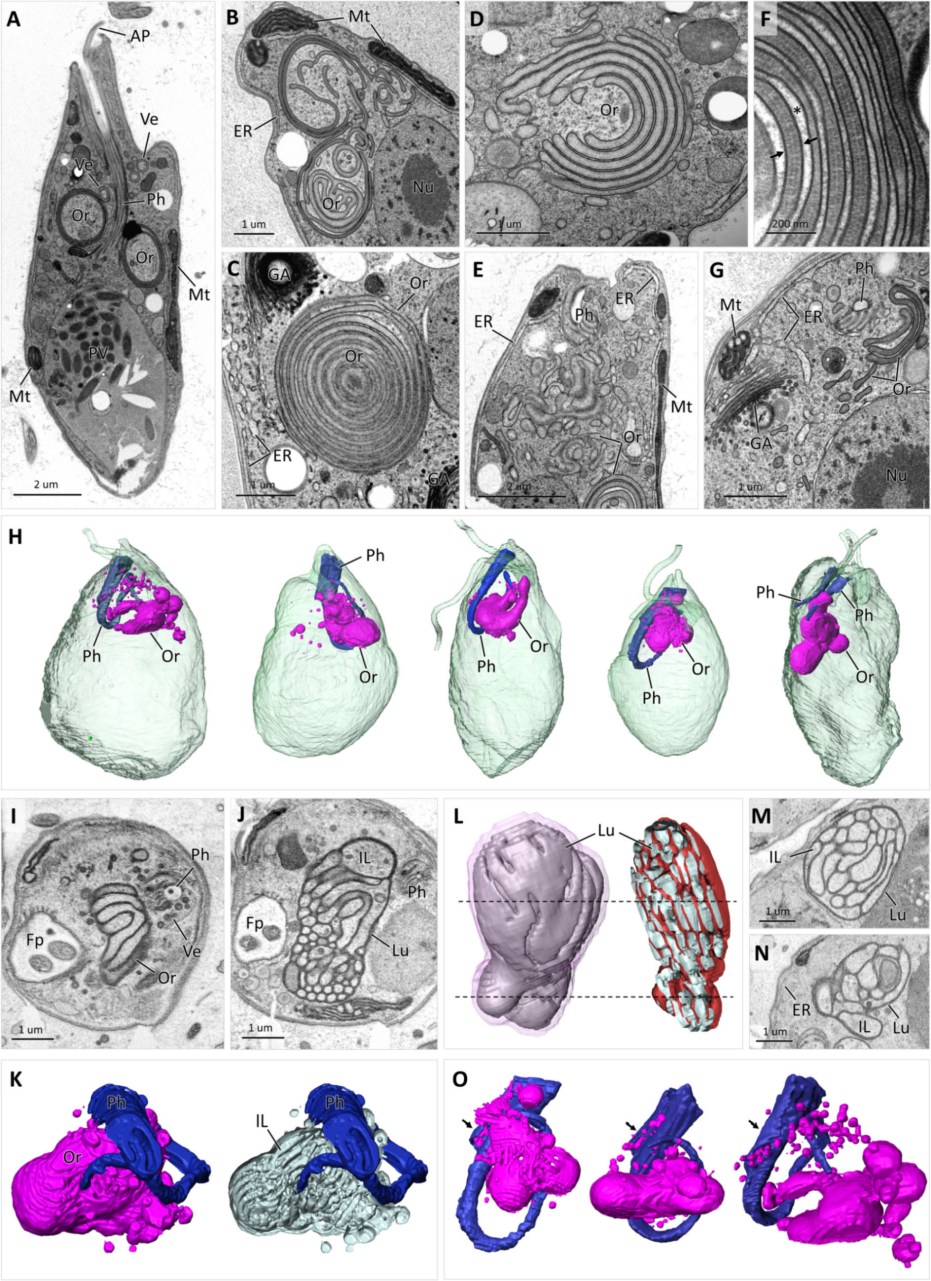
Morphology and ultrastructure of colv organelle. TEM images (**A–G**) of longitudinally sectioned cell (**A**) with two ring-shaped colv elements and multiple ring-shaped vesicles (Ve) in proximity to cytopharynx (Ph). Colv organelle (Or) composed of multiple long vesicles (**B**), concentric rings (**C**), stacked sacs (**D**), and elaborated reticulum (**E**). Close-up image (**F**) of colv concentric rings showing granular dark lumen between thicker membranes (arrows) and light intraluminal (IL) space (asterisk). Section (**G**) through colv tubules (Or), rough and smooth ER and GA. Five cells reconstructed from SBF-SEM data set (**H**) illustrate size and position of Ph and colv Or relative to the whole cell size. SBF-SEM images of cross-sectioned colv Or through top (**I**) and middle (**J**) part of organelle with honeycomb structure and corresponding 3D reconstruction (**K**) showing surface view (left) and IL tubules; note dark lumen (Lu), light IL, and multiple vesicles exiting Ph. 3D reconstruction (**L**) of IL space of colv Or showing outer IL sacs (left) and longitudinally sectioned sacs (right); dotted lines show section planes corresponding to SBF-SEM images **M** and **N**; note difference between density of Lu and ER membranes. 3D reconstruction (**O**) of Ph and associated colv Or in three different cells; note exiting Ve at peripheral microtubular band (arrows) of Ph.

The fluorescent staining of mitochondrial DNA (Fig. 2H and I) indicates that *L. vacuolata* houses a reticulated mitochondrion, a feature typical for other euglenozoans (25 – 28). The 3D reconstruction of five cells from the SBF-SEM data set showed that each cell features a single mitochondrion with no disconnected branches (Fig. 6A). The entire mitochondrion is invariably localized directly underneath the microtubular corset, except for a small portion of the organelle that branches off proximally to form a contact with the cytopharynx at its upward U-turn (Fig. 4M and 6B). Although the mitochondrion occupies a large subsurface area, in many regions, it varies to extremely thin and frequently collapses into two tightly juxtaposed membranes without discernible cristae or matrix in between (Fig. 6C, E and 9C, H[Fig F6]). These thin branches connect thicker bulging parts of the mitochondrion with electron-dense matrix containing prominent DNA aggregates (Fig. 6G) and complex cristae architecture. The most common type being very long lamellar cristae arranged parallel to each other and to the cell surface (Fig. 6D). Alternatively, straight or curved short lamellar cristae can be either parallel to each other or arranged irregularly (Fig. 6F), alternating with blob-like vesicular cristae (Fig. 6L and 7G). In addition, unusual circular cristae profiles ~300 nm in diameter were present in many TEM sections, which in the SBF-SEM data sets appeared as long curved cylinders (Fig. 6G; Movie S2). Analysis of SBF-SEM sections revealed that all of the above-mentioned cristae morphotypes were interconnected and present within a single cell.

The subspherical nucleus, 2.5 to 3.3 µm in diameter in non-dividing cells, is located in the anterior part of the cell, often peripherally (Fig. 6H, K, and L). It contains a prominent, usually central nucleolus and numerous condensed chromosomes (Fig. 6J through L). Examination of several dozens of cells from the SBF-SEM data set showed that each nucleus invariably contains a spherical structure resembling Cajal bodies (29), which are approximately 700 nm in diameter, localized peripherally (Fig. 6J; Movie S3). This electron-dense and fine-textured body is not closely associated with the nucleolus and heterochromatin. Occasionally, two such bodies were found within a single nucleus on the opposite sides of the nucleolus (Fig. 6K). However, their dual presence does not appear to be linked with cell division, as only a single such structure occurs in the elongated dividing nuclei.

A non-dividing cell usually contains two distinct, anteriorly localized Golgi bodies in the proximity of the nucleus and the FP (Fig. 4H and N), consisting of several flat or circular stacked cisternae (Fig. 6N and 7C, G). In the early stages of cell division, multiple smaller Golgi bodies can be found in a cell (Fig. 6I). Sacs of rough ER, continuous with the outer nuclear membrane (Fig. 6L), form an extensive network right beneath the microtubular corset or over the branches of the mitochondrion (Fig. 6M and 7E). However, peripheral lacunae, an expanded membrane-bound compartment typical for hemistasiids (9), are absent from *L. vacuolata*.

#### Ultrastructure of the colv, a novel membranous organelle

Examination of ultrathin TEM sections revealed the presence of unusual membranous structures in the anterior part of the cell, near the cytopharynx (Fig. 7A). The structures were composed of short and long flat vesicles (Fig. 7B), either solitary or forming stacks resembling the Golgi apparatus, and often arranged into simple rings, long tubules, and multilayered concentric structures of regularly spaced rings (Fig. 7B through E). The membranes surrounding them looked darker and thicker than those of the nucleus, Golgi apparatus, and ER (Fig. 7F and G), although solitary tubules and vesicles could not always be assigned to either of these organelles. When sections were contrasted with uranyl acetate and lead citrate, the intermembrane space (hereafter referred to as lumen) usually appeared darker than the cytoplasm and had a fine-grained or hair-like texture (Fig. 7F).

**Fig 8:**
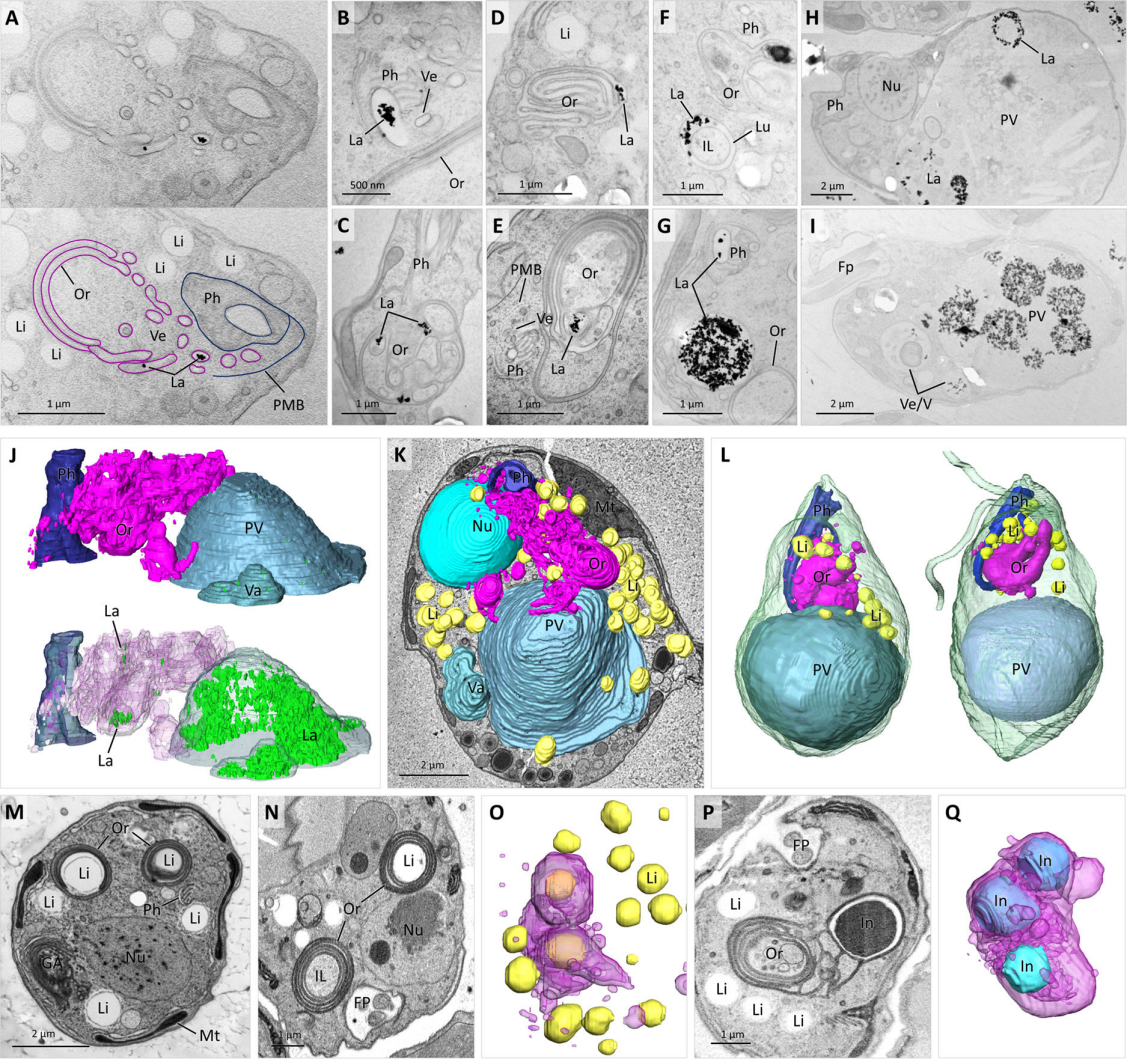
Association of colv organelles with cellular structures. TEM images (**A–I**) of cells fed with lanthanoid nanocrystals (La). In **A**, La-carrying vesicles exit cytopharynx (Ph) delimited by PMB and stack with colv organelle (Or); note presence of multiple lipid droplets (Li). La inside Ph lumen (**B**). Examples of colv Or carrying La inside its lumen (**D–E**). Ring-shaped (**F**) and round (**G**) Ve carrying La. Ring-shaped (**H**) and round (**I**) La pellets inside large PV and smaller vacuole (Va). 3D reconstruction of 2 µm-thick slice from array tomography data set (**J and K**) shows parts of Ph, reticulated colv Or originating from Ph and carrying La, and Va and PV filled with La (**J**) and top view on Ph, colv Or, PV, Li, and nucleus (Nu), superimposed on array tomography image (**K**) of obliquely sectioned cell with peripheral mitochondrion (Mt). Two cells reconstructed from SBF-SEM data set (**L**) showing position of Ph, Li, Or, and PV. TEM image (**M**) illustrating Li encircled with colv Or elements. SBF-SEM image (**N**) of Li completely enclosed within colv Or, and similar Or element with light intraluminal space inside. 3D reconstruction (**O**) of colv Or and Li from cell depicted in **N**. SBF-SEM image (**P**) illustrating dark inclusion (In) completely enclosed within colv Or. 3D reconstruction (**Q**) of colv Or with two enclosed and one external In.

Analysis of dozens of cells by SBF-SEM showed that this organelle, named here the colv (Center for Organization of Layered Vesicles), is present in every cell, albeit its shape, size, and internal structure vary (Fig. 7H). The 3D reconstruction revealed that the individual elements seen in the ultrathin sections were usually connected into an elaborated reticulum (Fig. 8K) or a single compact organelle that included multiple circular and tubular elements bound by a common membrane (Fig. 7O, 8C, E, and P). The organelle was always associated with the cytopharynx and occupied a significant portion of the anterior part of the cell (Fig. 7H; Movie S3). Heavy-metal contrasting used for preparation of the SBF-SEM samples resulted in intense staining of the organellar membranes, making them clearly distinguishable from the ER (Fig. 7I, M, and N; Movie S3). The organelle often takes a honeycomb appearance, where the walls are formed by interconnected double membranes with a dark lumen between them, while the intraluminal space is composed of portions of cytoplasm arranged into bundles of long discrete sacs and tubes (Fig. 7I through N; Movie S3).

Multiple simple small vesicles or larger double-membrane ring-shaped vesicles were additionally associated with the organelle. By examining SBF-SEM serial sections, the origin of the vesicles could be traced to the cytopharynx, where they pinched off the pharyngeal lumen and seemingly fused with the organelle (Fig. 7I, O and 8K). To confirm the association of the organelle with the cytopharynx-derived vesicles, live cells were incubated with lanthanoid nanocrystals for 1 h, and then were processed for TEM by high-pressure freezing technique (Fig. 8A through I). The nanocrystals were readily visible inside the pharyngeal lumen (Fig. 8B and G) and cytopharynx-derived vesicles (Fig. 8A), including the ring-shaped vesicles, in which the crystals were found inside the colv’s lumen (Fig. 8F).

Moreover, the nanocrystals were also found in long tubular vesicles that formed stacks with the organellar sacs (Fig. 8A) and established themselves directly inside the organellar matrix (Fig. 8C through E). However, they were never seen inside the colv’s intraluminal space and were associated neither with other organelles nor the cytoplasm. Alternatively, vesicles carrying nanocrystals were routed to the posterior vacuole, where crystals often retained round- or ring-shaped appearance of the vesicles (Fig. 8H and I). Smaller vesicles evidently gradually merged into larger vesicles (Fig. 8G), which eventually fused with the lysosomes, producing digestive vacuoles (Fig. 2K). Analysis of ultrathin serial sections obtained by array tomography showed that when the colv is assembled into a reticulum, the lanthanoid crystals are capable of moving within its lumen and are eventually purged into the posterior vacuole (Fig. 8J).

The anterior half of the cell near the colv is usually packed with small cytoplasmic lipid droplets (Fig. 8K and L), which were identified by BODIPY staining (Fig. 2J). Approximately 25% cells in the SBF-SEM data set contained one to several lipid droplets inside the colv, while in many other cells, individual sacs were stacked around the droplets (Fig. 8M and N). The 3D reconstruction of these cells revealed that lipid droplets were often completely enclosed within the organelle (Fig. 8O). Less frequently, dark osmiophilic inclusions were contained in the colv’s intraluminal space as well (Fig. 8P and Q). These large, always strictly round-shaped granules, presumably serving as reserve material, were up to 8 µm in diameter and were present in the cytoplasm of most cells.

**Fig 9 F9:**
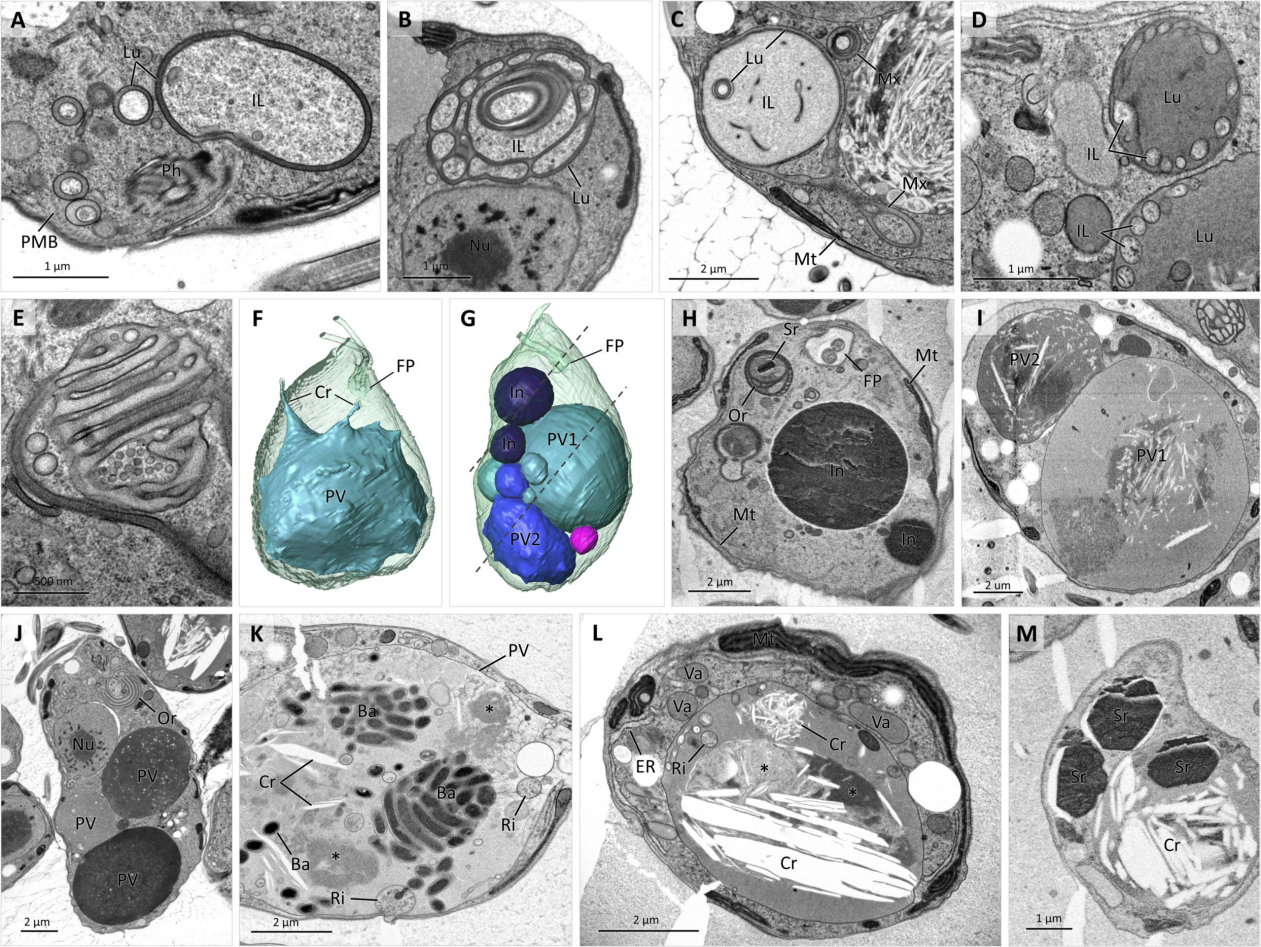
Morphological plasticity of colv organelle and vacuoles. TEM images of colv organelle (Or) and associated vesicles (**A–E**). Multiple ring-shaped vesicles (**A**) exiting cytopharynx (Ph), directed by PMB; note dark lumen (Lu) between membranes and light portions of cytoplasm (IL). Or of alveolate appearance (**B**) with dark Lu and light intraluminal space. Or with IL devoid of ribosomes carrying ring-shaped vesicle (**C**). Or transforming into dark vacuole by expanding its Lu (**D**). Microvesicular appearance of Or (**E**). 3D reconstruction of two cells (**F and G**) carrying a single massive PV with protruding crystals (Cr) in **F** and two PVs with different content accompanied by smaller vacuoles and dark reserve inclusions (In); dotted lines show section planes corresponding to SBF-SEM images **H** and **I** small; purple Va carry undigested pellet (not shown). In **H**, note SrSO_4_ crystal (Sr) enclosed inside Or. In **I**, light PV1 and dark PV2 contain long needle-like crystals. Longitudinal TEM section (**J**) through three PV. TEM (**K and L**) and SBF-SEM (**M**) images of posterior vacuoles containing bacteria (Ba), vesicles with ribosomes (Ri), light Cr, dark Sr, and undigested pellets (asterisk).

Simple spherical vesicles originating from the cytopharynx are apparently membrane-enclosed portions of the cytopharyngeal lumen carrying nutrients (Fig. 8A). The double-membrane ring-shaped vesicles are likely formed by encircling portions of the cytoplasm similarly to autophagosomes (30), whereas, as suggested by tracing with nanocrystals, the nutrients are sequestered into the electron-dense lumen localized between the membranes. Internalized portions of the cytoplasm change their texture and electron density immediately after exiting the cytopharynx and become completely devoid of ribosomes (Fig. 9A [Fig F9]. Such lighter-stained portions of the cytoplasm apparently either evolve into the intraluminal space of the colv (Fig. 7A, B, F and 9B) or are expanded and degraded, eventually turning into vacuoles with electron-lucent content (Fig. 9C). Of note, ring-shaped vesicles encircling intact cytoplasm were also commonly seen. Alternatively, the organellar lumen can expand, turning parts of the colv into vacuoles with electron-dense content (Fig. 9D). In addition, the colv often transforms into multivesicular body-like structures (Fig. 9E).

#### Posterior vacuole

The posterior vacuole is present in all studied cells, yet its volume dramatically varies (Fig. 8L and 9G) and is often accompanied by smaller vacuoles and reserve granules that together occupy most of the cell’s volume (Fig. 9F through H and J). The interior of these vacuoles ranges from clear homogeneous material of variable electron density (Fig. 9J) to a complex assortment of undigested food pellets, small and large needle-shaped organic crystals, mineral (SrSO_4_) crystals (17), and portions of cytoplasm with intact ribosomes (Fig. 9I and K through M). When the medium is supplemented with bacteria, multiple round pellets of intact undigested bacteria, as well as solitary bacterial cells can be seen inside the posterior vacuole (Fig. 7A and 9L). The organic needle- and spindle-like crystals (Fig. 5N and 9I, K through M) that were present in most examined cells sometimes exceeded 10 µm in length and stretched both the vacuolar and plasma membranes, even protruding beyond the cell surface (Fig. 9F).

## DISCUSSION

This study presents the first 3D reconstruction of a diplonemid cell, which, when complemented with high-resolution TEM analysis, provides not only an accurate mapping of structures and organelles common for these protists but also identifies previously unknown or unnoticed peculiarities of diplonemid ultrastructure. Among these is the unusual architecture of a single mitochondrion featuring extensive thin branches and sheets of juxtaposed membranes with no matrix and cristae between them. We also document in *L. vacuolata* a unique feature represented by a complex array of interconnected vesicular, and short and long lamellar cristae that are additionally arranged into long cylinders. In diplonemids, cristae are usually organized into parallel flat lamellae of variable length (9), whereas other euglenozoans possess discoidal cristae or lack them entirely (31, 32). We also acknowledge the common presence of small hair-coated vesicles that pinch off the flagellar pocket into the extracellular space. These have previously been observed in diplonemids (8, 14), but their role has never been discussed. Since diplonemids were often described as predators of larger planktonic organisms such as diatoms, dinoflagellates, and copepods, or opportunistic parasites of macroscopic invertebrates such as crabs, clams, and lobsters (9), extracellular microvesicles may carry adhesins, toxins, digestive enzymes, or immunomodulators ensuring adhesion, penetration, and persistence inside host/prey cells and tissues, or additionally take part in cell-to-cell communication. Similar behavior is common among taxonomically unrelated parasitic and pathogenic protists (33 – 37).

Moreover, the findings presented here give insights into the biology and cellular architecture of other euglenozoans. Specifically, a detailed description of the ultrastructure of cell division is available for only three euglenozoans—parasitic *Trypanosoma brucei* (25) and *T. cruzi* (38) and photosynthetic *Euglena gracilis* (39). Yet, these few species do not represent adequately the overall diversity of mostly biflagellate phagotrophic euglenozoans with complex feeding apparatuses. The knowledge of cell division for a handful of other euglenids as well as kinetoplastids and only one diplonemid is mostly restricted to the ultrastructure of the mitotic nucleus, replication of pellicular strips and microtubules, and duplication of basal bodies and flagellar roots (40 – 45).

Here, we identified in a diplonemid that replication is accompanied by disintegration of its flagellar and feeding apparatuses at the onset of cell division and their subsequent assembly in daughter cells. Furthermore, re-examination of SEM samples showed that two other diplonemids (*P. papillatum* and *Lacrimia lanifica*) possess two pairs of short flagella during cell division (data not shown). The reduction of maternal flagella has been sporadically reported in several euglenozoans, including *E. gracilis* (39, 46), *Cyclidiopsis acus* (47), and *Hemistasia phaeocysticola* (48), but the mechanism of this process has not been described. In model organisms such as *Chlamydomonas reinhardtii*, the flagellar shortening occurs *via* gradual depolymerization of the axonemal microtubules occurring at the flagellar tip (49), whereas in the mammalian cells, both detachment of the entire axoneme and depolymerization take place (50). Neither seems to operate in *L. vacuolata*, which shreds its axonemes at multiple sites, representing a previously unknown mechanism of flagella reduction. So far, it is unclear whether the axonemal fragments are resorbed and recycled or discarded into the extracellular medium. Similarly, at least two phagotrophic euglenids, *Entosiphon sulcatum* (43, 51) and *Ploeotia costata,* (44) and the trypanosomatid *T. cruzi* (38) also undergo degradation of their cytopharynxes prior to cell division. Hence, disintegration of the feeding apparatus might be a general trait of euglenozoans, while an extensive shortening of the maternal flagellum during proliferative cell division apparently does not occur in trypanosomatids (25, 52, 53).

The 3D reconstruction of this diplonemid also identified a feature of division that is unique so far among Euglenozoa—establishment of a septum along nearly the entire length of a dividing cell before the onset of cytokinesis. The septum is formed by two symmetric transverse invaginations of the microtubular corset, largely detached from the plasma membrane, which serves to partition organelles into the daughter cells. In contrast, the segregation of organelles occurs simultaneously with cytokinesis in *T. brucei* (25, 54), *T. cruzi* (55), *E. sulcatum* (43), and *E. gracilis* (56), and their cytoplasm remains largely connected as the cleavage furrow progresses separating the daughter cells. A similar septum has not been described in other euglenozoans, although the cleavage furrow of *C. acus* was associated with additional microtubules (47). Among diplonemids, at least *L. vacuolata* produces both equal- and unequal-sized daughter cells, which also may receive different numbers of large posterior vacuoles. Furthermore, the position of flagellar-cytopharyngeal-nuclear complexes is not stringent, as these either remain adjacent to the septum or migrate to the opposite poles of a dividing cell. An unequal and asymmetric division has so far been reported in *T. brucei* (57), but future reconstructions of additional euglenozoans hold the promise to elucidate whether this is a common trait for this group.

Our study provides the first insight into the development, structure, and function of an unusual organelle, named here the colv, which is a part of an elaborate digestion-related endomembranous system that includes various vesicles and vacuoles. It exists in parallel to the conserved set of eukaryotic core membrane trafficking machinery (34) and shares morphological similarities with the Golgi apparatus and the ER (58). This organelle docks the endocytic cytopharyngeal membrane-derived vesicles, transports enclosed nutrients within its lumen throughout the cell, forms stacks resembling those of the Golgi apparatus, transforms into microvesicular-like bodies, and is likely involved in metabolism of reserve lipids, as judged by their frequent association with the colv. Differential routing of vesicles, traced with lanthanoid nanoparticles, suggests that these vesicles are pre-sorted immediately following budding off the pharyngeal lumen. Thus, certain pre-sorted nutrients delivered to the organellar lumen by the endocytic vesicles might be used directly for lipid synthesis, bypassing the digestive vacuoles. This represents an additional or alternative pathway of lipid droplet biogenesis, which normally takes place within the ER membrane (59). Conversely, the colv might be involved in the opposite process, namely lipophagy, which entails autophagocytosis of lipid droplets (60). Furthermore, the colv transforms its parts into various vacuoles by expanding either matrix or luminal space that likely creates vacuolar compartments with selected cargo for biosynthesis of inclusions, among which are electron-dense, possibly reserve granules, celestite crystals, and electron-lucent organic crystals (17). Only small LysoTracker-stained dots were present around the cytopharynx, indicating that some parts of the colv might be acidified, but not its entire volume.

The large posterior vacuole appears to be the final destination of the aforementioned plethora of vacuoles and vesicles. Although in other diplonemids such vacuoles were considered “food vacuoles” (3, 7, 48, 61), our results suggest that in *L. vacuolata* and likely in related flagellates, these are not digestive vacuoles, although undigested food particles such as bacterial pellets and lanthanoid crystals end up here. Instead, as LysoTracker staining indicated, food digestion occurs in smaller acidic vacuoles, which are eventually emptied into the non-acidified posterior vacuole. This large cellular structure has a diverse content of abundant organic and mineral crystals, portions of the cytoplasm, lamellar structures reminiscent of the autophagosome cargo (62), as well as various granules and small vesicles. With one notable exception (63), euglenozoans, including *L. vacuolata*, lack a morphologically prominent cytoproct. Nevertheless, the content of the posterior vacuole might be eventually extruded from the cell through expulsion of the entire vacuole, not yet observed in *L. vacuolata*, but documented for other diplonemids (6, 23). Alternatively, like fungal vacuoles, the posterior vacuole can serve as a homeostatic organelle, which stores minerals, osmotic solutes, nutrients, and waste products (64).

Only *L. lanifica*, the closest relative of *L. vacuolata*, featured a similar colv organelle (8), albeit less complex. Nevertheless, the cytoplasm of most diplonemids is rich in similar spherical, elongated, crescent-shaped, and ring-shaped vesicles emerging from the cytopharynx (8, 9, 14, 21, 23, 48, 61), but these are not organized into concentric rings, stacks, and honeycomb structures like in *L. vacuolata*. Further 3D reconstructions of these species might elucidate whether vesicles observed in 2D images are organized into other structures, such as an elaborated reticulum, and associated with various types of vacuoles. In *L. vacuolata*, the endocytic vesicles exit at a very narrow region of its unusually long cytopharynx, otherwise mostly filled with cytoplasm, while several other diplonemids carry the luminal space for most of its length (9, 21). The function of the lumen-free region of the cytopharynx is not apparent, although it seems to be involved in microvesicular transport, while the microtubules radiating from its terminus may tether the cytopharynx to other cytoskeletal elements, cell structures, and organelles.

The observation of a new organelle represents an intriguing discovery and demonstrates the exciting potential from investigating poorly studied protist groups. A better understanding of the colv’s function and endocytosis in diplonemids will require coordinated future efforts, including the establishment of a range of molecular data and tools, but holds the promise to broaden our understanding of eukaryotic molecular biology.

## MATERIALS AND METHODS

### Cultivation, DNA isolation, amplification, and sequencing

Clonal axenic cell culture of *Lacrimia* sp. YPF1808 was established by isolating a single cell from a plankton net sample (25 µm mesh size) collected at the port of JAMSTEC headquarters in Yokosuka, Japan (35.3194°N; 139.6507°E) as described previously and routinely grown in Hemi medium at 13°C (7, 8). For all analyses, cells were harvested by centrifugation at 3,000 *g*. DNA isolation, PCR amplification, and 18S rRNA gene amplicon sequencing were carried out as in reference (8).

### Phylogenetic analysis

Data set for the phylogenetic reconstruction was adapted from reference (23). The resulting data set comprising of 86 diplonemid and 10 kinetoplastid 18S rRNA sequences was aligned using the MAFFT v7.453 under the localpair algorithm and ambiguously aligned regions with large proportions of gaps were manually removed. A maximum likelihood phylogeny was reconstructed with IQTree 2.1.2 (65) under the General Time Reversible model with the base frequencies estimated from the alignment and four relaxed rate categories (GTR + F + R4). This particular model was found as best fitting by the phylogenetic model finder implemented in IQTree according to both corrected Akaike and Bayesian information criteria. Branching robustness was estimated using the thorough non-parametric bootstrap support estimated from 500 replicates in IQTree (-b 500 flag) as well as Bayesian posterior probabilities. For the latter, two Markov chains were run in Phylobayes 4.1 (66) until they converged (maximum observed discrepancy <0.1 and effective samples size of model parameters >100) under the GTR + C20 model.

### Light microscopy and fluorescence staining

Visualizing mitochondria with 100 nM or 500 nM MitoTracker Red CMXRos, 100 nM or 200 nM MitoTracker Green FM, 2 nM, 6 nM, 10 nM, or 20 µM DiOC_6_(3) membrane potential dye was unsuccessful. Therefore, mitochondria were *in vivo* stained with 500 nM of DNA-specific SYTO24 dye as described elsewhere (14). For visualizing DNA, cells were fixed with 2% OsO_4_ in seawater for 15 min, rinsed of the fixative, air-dried on glass slides, and mounted in ProLong Gold antifade reagent (Life Technologies) containing 4,6-diamidino-2-phenylindole. Lysosomes were *in vivo* stained with 50 nM of LysoTracker (Life Technologies) diluted in Hemi medium for 30 min. Lipids were stained *in vivo* with BODIPY 493/503 fluorescent dye (Thermo Fisher). Microscopy slides were observed under an Olympus BX53 microscope equipped with differential interference contrast or Zeiss AxioPlan 2 fluorescence microscope. Images and videos were captured with a DP72 microscope digital camera at 1,600 × 1,200-pixel resolution using CellSens software v. 1.11 (Olympus) and processed in GIMP v. 2.10.14, Irfan View v. 4.54, and Image J v. 1.53t software.

### Array tomography

Lanthanoid nanocrystals were synthesized according to reference (67) by mixing 7.5 mL of 0.1 M La(NO_3_)_3_·6 H_2_O, 2.5 mL of 0.01M TbCl_3_, and 35 µL polyethylenglycol methyl ether amine (mPEG amine, Mn 500), followed by dropwise adding of 2.5 mL of 0.32M NH_4_F solution (all from Sigma-Aldrich) at room temperature (RT) under continuous stirring. The reaction solution was further stirred at 150°C for 4 h. Synthesized nanocrystals were centrifuged using Amicon 100K filter units (Merck Millipore) and washed three times with ethanol and deionized water. The sizes of LaF_3_:Tb^3+^ nanocrystals ranged from 14 nm to 80 nm.

A culture grown in Hemi medium was incubated with lanthanoid nanocrystals for 2 h prior to harvesting the cells by centrifugation. Cell pellets were transferred to specimen carriers and immediately frozen in the presence of 20% wt/vol bovine serum albumin solution using a high-pressure freezer Leica EM ICE (Leica Microsystems). Freeze substitution was performed in the presence of 2% OsO_4_ diluted in 100% acetone at −90°C for 96 h. Specimens were warmed to −20°C at a step of 5 °C/h and, subsequently, to 3°C at a 3°C/h step. At RT, samples were washed in acetone and infiltrated with 25%, 50%, 75% acetone/resin mixture for 1 h at each step. Finally, samples were infiltrated in 100% Epon resin, Embed-812 (EMS), and polymerized at 60°C for 48 h. Ultrathin (70 nm) sections were collected on a silicon wafer, which was glued to the SEM pins using double carbon tape, post-stained with uranyl acetate for 45 min and lead citrate for 20 min, and carbon coated. Array sections were imaged using Apreo SEM and MAPS software (Thermo Fisher). Serial images were acquired using immersion mode: accelerating voltage 2.5 keV, probe current 0.2 nA, working distance 5.6 mm, resolution 4 nm, slice thickness 70 nm, dwell time per pixel 3 µs. The image data were processed by Microscopy Image Browser v. 2.8331 and Amira v. 2022.1. The materials were visualized in surface rendering mode and smoothen with constrained or unconstrained smoothening algorithms. Movies were prepared in Image J v. 1.53t software.

### SBF-SEM

The sample preparation by high-pressure freezing technique followed the protocol for Array Tomography section sample preparation (culture without lanthanoids was used). After freeze substitution, the samples were stained at RT with <1% thiocarbohydrazide in 100% acetone for 1.5 h, 2% OsO_4_ in 100% acetone for 2 h (all at RT), and 1% uranyl acetate in 100% acetone overnight at 4°C. After every staining step, the samples were washed three times with 100% acetone for 15 min, infiltrated with 25%, 50%, 75% acetone/resin mixture for 2 h at each step, and finally infiltrated by EMS 812 hard resin plus overnight and polymerized at 62°C for 48 h. Resin-embedded blocks were trimmed and imaged using Apreo SEM equipped with the VolumeScope (Thermo Fisher). Serial images were acquired at 3.5 keV, 50 pA, 40 Pa with a resolution of 6 nm and 100 nm slice thickness, and dwell time per pixel of 4 µs. The image data were processed as described above.

### SEM and TEM

TEM samples were prepared by high-pressure freezing as described in reference (15) and observed with the JEOL-1010 and JEOL-1400 TEMs at accelerating voltage of 80 kV and 120 kV, respectively. For SEM, pellets were fixed with 2% OsO_4_ in seawater, processed as described previously (8), and observed with a JEOL JSM-7401-F microscope at an accelerating voltage of 4 kV.

## Data Availability

The near full-length 18S rRNA gene sequence is deposited in GenBank under the accession number OR094903.

## SUPPLEMENTAL MATERIAL

Text S1 mbio.01921-23-s0001.docx Taxonomic summary.

Table S1 mbio.01921-23-s0002.xlsx 18S rRNA similarity values among representatives of Diplonemidae and Hemistasiidae relevant for this study.

Movie S1 mbio.01921-23-s0003.mpg Live cells of *L.vacuolata* sp. nov. viewed with differential interference contrast.

Movie S2 mbio.01921-23-s0004.mpg Sequential SBF-SEM images illustrating position of flagellar roots, cytopharynx, and mitochondrial cristae arranged in long cylinders (labeled as tubules).

Movie S3 mbio.01921-23-s0005.mpg Sequential SBF-SEM images through cell anterior illustrating cross-sectioned cytopharynx, spherical nuclear body, peripheral mitochondrion, and colv organelle.

ASM does not own the copyrights to Supplemental Material that may be linked to, or accessed through, an article. The authors have granted ASM a non-exclusive, world-wide license to publish the Supplemental Material files. Please contact the corresponding author directly for reuse.
