# Supplementary material for: Ultrastructure and 3D reconstruction of a diplonemid protist (Diplonemea) and its novel membranous organelle
Source: mBio. 2023 Sep 22;14(5):e01921-23. doi: 10.1128/mbio.01921-23 (PMC10653844; doi:10.1128/mbio.01921-23)
Supplement: Text S1 — Taxonomic summary. [file mbio.01921-23-s0001.docx]

**TAXONOMIC SUMMARY**

Phylum Euglenozoa Cavalier-Smith 1981, emend. Simpson 1997; class Diplonemea Cavalier-Smith 1993, emend. Simpson 1997; genus *Lacrimia* Tashyreva, Prokopchuk, Horák and Lukeš 2018.

*Lacrimia vacuolata* sp. nov. Tashyreva and Lukeš 2023

Species designated based on discrepancy in morphology and 18S rRNA gene with *Lacrimia lanifica* (92.9% similarity).

Description. Teardrop-shaped cells with one or several large posterior vacuoles and narrowed anterior ends; smooth surface; distinguished from *L. lanifica* by larger size – 10.1 to 23.4 µm in length (18.1±3.1 µm; N=50) and 5.9 to 12.9 µm in width (9.5±2 µm; N=50), more complex colv organelle, and larger number of posterior vacuoles; subapical FP, two morphologically identical flagella with heteromorphic PFR arising from parallel basal bodies, one to two times of body length, dorsal flagellum one third longer than ventral flagellum; fast swimming mediated by dorsal flagellum forming anterior loop and ventral flagellum loosely twisted along body, gliding along surfaces and metaboly; do not differentiate into other life stages; extrusomes and endosymbiotic bacteria absent; apical cytostome associated with narrow lip-like papilla; cytopharynx forming a loop; peripheral nucleus 2.5 to 3.3 µm in diameter, situated in anterior half; single peripheral reticulated mitochondrion with long lamellar and vesicular cristae.

Etymology. The species name denotes the presence of multiple large posterior vacuoles and reserve granules.

Type strain. *Lacrimia vacuolata* YPF1808.

Type locality. Surface coastal water of the Sea of Japan at the port of JAMSTEC headquarters in Yokosuka, Japan (35.3194°N; 139.6507°E).

GenBank accession number: OR094903.

Hapantotypes are a block of resin-embedded cells for transmission electron microscopy and ethanol-fixed cells deposited at the protozoological collection of the Institute of Parasitology, Biology Centre, Czech Academy of Sciences, České Budějovice, Czech Republic under no. IPCAS Prot 76.
